# Identification of potential classes of glycoligands mediating dynamic endothelial adhesion of human tumor cells

**DOI:** 10.1093/glycob/cwad061

**Published:** 2023-07-24

**Authors:** Sarah Starzonek, Hanna Maar, Stefan Mereiter, Vera Freytag, Marie-Therese Haider, Kristoffer Riecken, Yen-Lin Huang, Francis Jacob, Daniel Wicklein, Udo Schumacher, Tobias Lange

**Affiliations:** Institute of Anatomy & Experimental Morphology, University Medical Center Hamburg-Eppendorf, Martinistrasse 52, 20246 Hamburg, Germany; Institute of Anatomy & Experimental Morphology, University Medical Center Hamburg-Eppendorf, Martinistrasse 52, 20246 Hamburg, Germany; Institute of Anatomy I, University Hospital Jena, Teichgraben 7, 07743 Jena, Germany; Comprehensive Cancer Center Central Germany (CCCG), 07743 Jena, Germany; Institute of Molecular Biotechnology, Austrian Academy of Sciences, Dr. Bohr-Gasse 3, 1030 Vienna, Austria; Institute of Anatomy & Experimental Morphology, University Medical Center Hamburg-Eppendorf, Martinistrasse 52, 20246 Hamburg, Germany; Institute of Anatomy & Experimental Morphology, University Medical Center Hamburg-Eppendorf, Martinistrasse 52, 20246 Hamburg, Germany; Research Department Cell and Gene Therapy, Department of Stem Cell Transplantation, University Medical Center Hamburg-Eppendorf, Martinistrasse 52, 20246 Hamburg, Germany; Ovarian Cancer Research, University Hospital Basel and University of Basel, Hebelstrasse 20, 4031 Basel, Switzerland; Ovarian Cancer Research, University Hospital Basel and University of Basel, Hebelstrasse 20, 4031 Basel, Switzerland; Institute of Anatomy & Experimental Morphology, University Medical Center Hamburg-Eppendorf, Martinistrasse 52, 20246 Hamburg, Germany; Department of Anatomy and Cell Biology, University of Marburg, Robert-Koch-Strasse 8, 35037 Marburg, Germany; Institute of Anatomy & Experimental Morphology, University Medical Center Hamburg-Eppendorf, Martinistrasse 52, 20246 Hamburg, Germany; Medical School Berlin, Leipziger Platz 10, 10117 Berlin, Germany; Institute of Anatomy & Experimental Morphology, University Medical Center Hamburg-Eppendorf, Martinistrasse 52, 20246 Hamburg, Germany; Institute of Anatomy I, University Hospital Jena, Teichgraben 7, 07743 Jena, Germany; Comprehensive Cancer Center Central Germany (CCCG), 07743 Jena, Germany

**Keywords:** E-selectin, HUVEC, laminar flow adhesion assay, sialylated Lewis antigens, tumor cell adhesion

## Abstract

One critical step of metastasis formation is the extravasation of circulating tumor cells from the bloodstream. This process requires the dynamic interaction of cell adhesion molecules like E-selectin on endothelial cells with carbohydrate ligands on tumor cells. To characterize these glycans in a comprehensible approach, the rolling, tethering, and firm adhesion of nine human tumor cell lines on human umbilical vein endothelial cells was analyzed using laminar flow adhesion assays. The tumor cell lines were grouped into three subsets by their canonical E-selectin ligand status (sialyl-Lewis A and X +/+, −/+, −/−) and their adhesiveness was compared after enzymatic, pharmacologic, chemical treatment or antibody blockade of the tumor cells or endothelial cells, respectively. Tumor cells were also screened regarding their glycosyltransferase expression profile. We found that although E-selectin and terminal α2,3-sialic acid largely determined firm adhesion, adhesive events did not exclusively depend on the presence of sialyl-Lewis A and/or sialyl-Lewis X. Nevertheless, two of the three sialyl-Lewis A/X−/− tumor cells additionally or fully depended on vascular cell adhesion molecule-1 for firm adhesion. The significance of *O*-GalNAc- and *N*-glycans for adhesion varied remarkably among the tumor cells. The sialyl-Lewis A/X+/+ subset showed glycoprotein-independent adhesion, suggesting a role of glycolipids as well. All sialyl-Lewis A/X−/− tumor cells lacked FUT3 and FUT7 expression as opposed to sialyl-Lewis A/X+/+ or −/+ cell lines. In summary, the glycans on tumor cells mediating endothelial adhesion are not as much restricted to sialyl-Lewis A /X as previously assumed. The present study specifically suggests α2,3-linked sialic acid, *O*-GalNAc glycans, glycosphingolipids, and FUT3/FUT7 products as promising targets for future studies.

## Introduction

Distant metastases and their clinical consequences are the major cause of cancer-related deaths worldwide (Chaffer and Weinberg 2011; Joosse et al. 2015; Dillekås et al. 2019). The formation of metastases at distant sites is preceded by a sequence of events including the interaction of circulating tumor cells (CTCs), which had originally detached from the primary tumor (PT), with endothelial cells (ECs) lining the vascular wall at the target site of the future metastasis. This dynamic endothelial adhesion and the subsequent extravasation of CTCs rescues the tumor cells (TCs) from the adverse conditions within the bloodstream (mechanical shear forces, immune cell attack, anoikis) that limit the survival time of CTCs (Joosse et al. 2015; Dasgupta et al. 2017; Strilic and Offermanns 2017). Extravasation is hence proposed to be one of the most crucial steps during hematogenous metastasis formation (Reymond et al. 2013; Dasgupta et al. 2017; Lambert et al. 2017; Strilic and Offermanns 2017). It is assumed that less than 0.01 percent of CTCs survive long enough to produce metastases (Joyce and Pollard 2009; Strilic and Offermanns 2017).

The adhesive interactions between TCs and ECs are supposed to mimic those of leukocytes and ECs during inflammation and homing so that different ligands and receptors like endothelial selectins, integrins, and members of the immunoglobulin superfamily have to be considered (Strell and Entschladen 2008; Reymond et al. 2013; Sökeland and Schumacher 2019; Tvaroška et al. 2020). Genetic knockout of the endothelial (E- and P-) selectins as well as knockdown of the E-selectin ligand carrier glycoprotein CD44 reduce spontaneous metastasis formation of human TCs in xenograft mouse models. Likewise, pharmacologic inhibition of E-selectin expression shows anti-adhesive und thus anti-metastatic efficacy on TCs with low-affinity E-selectin ligands (Köhler et al. 2010; Stübke et al. 2012; Gebauer et al. 2013; Wicklein et al. 2013; Heidemann et al. 2014; Lange et al. 2022).

E-selectin promotes metastasis formation by interacting with α1,3/α1,4-fucosylated and α2,3-sialylated terminal glycan residues on glycoproteins, glycolipids, or proteoglycans expressed on CTCs (Varki et al. 2009; Trinchera et al. 2017; Tvaroška et al. 2020). The tetrasaccharide sialyl-Lewis X (sLeX, CD15s: Neu5Acα2,3Galβ1,4(Fucα1,3)GlcNAc-R) and its isomer sialyl-Lewis A (sLeA, CA 19–9: Neu5Acα2, 3Galβ1,3(Fuc-α1,4)GlcNAc-R) are well-described E-selectin-binding glyco-epitopes and are commonly expressed on TCs according to the current literature (Khatib et al. 2005; Reymond et al. 2013; Borsig 2018; Starzonek et al. 2020; Ganesh et al. 2021). Several glycoproteins carrying the canonical selectin ligands sLeA and sLeX like PSGL-1, ESL-1, CD24, CD43, CD44, MAdCAM-1, LAMP-1, LAMP-2, etc., have been identified so far (St Hill 2011; Läubli and Borsig 2019). Increased selectin ligand expression on TCs correlates with enhanced metastasis and poor prognosis of cancer patients (Läubli and Borsig 2010; Natoni et al. 2016; Borsig 2018). Despite the many different types of E-selectin ligands on human TCs described, there is no comprehensive picture yet.

Besides E-selectin, vascular adhesion receptors of the immunoglobulin superfamily such as intercellular adhesion molecule-1 (ICAM-1) and vascular cell adhesion molecule-1 (VCAM-1) are also involved in endothelial adhesion of CTCs. VCAM-1 has been reported to bind to the α4 integrins “very late antigen 4” (VLA-4, integrin α_4_β_1_, or CD49d/CD29) and α_4_β_7_ whereas ICAM-1 is recognized by the β2 integrins “lymphocyte function-associated antigen 1” (LFA-1, α_L_β_2_, or CD11a/CD18) and αM (Mac-1, α_M_β_2_, or CD11b/CD18) (Schlesinger and Bendas 2015; Kong et al. 2018; Bui et al. 2020).

The expression of vascular CAMs like E-selectin, ICAM-1, and VCAM-1 is not consistently regulated throughout all organs. Human skin and bone marrow endothelium show a constitutive expression of E-selectin and VCAM-1 (Schweitzer et al. 1996; Läubli and Borsig 2010; Ales and Sackstein 2023), whereas in other organs like the lung, expression of E-selectin, ICAM-1 and VCAM-1 is cytokine-regulated in ECs (Haraldsen et al. 1996). For the TC adhesion to the endothelium and subsequent transmigration into the adjacent tissue, ECs need activation first in most vessels. It is assumed that this distant endothelial activation is caused by systemic effects of the tumor disease, either through CTCs itself or trough triggered microenvironmental responses in distant tissues (McAllister and Weinberg 2014). For instance, it was shown that the influx of metastatic TCs into the hepatic microvasculature can trigger Kupffer cells located in sinusoidal vessels to produce the proinflammatory cytokines interleukin 1 alpha (IL1a) and tumor necrosis factor alpha (TNFa), leading to increased expression of E-selectin, VCAM-1, and ICAM-1 on sinusoidal ECs (Auguste et al. 2007; Borsig 2018). Furthermore, co-culture of the human lung adenocarcinoma cells CL1–5 with HUVECs caused an upregulation of VCAM-1 and ICAM-1 (Cheng et al. 2017). Following the activation by tumor- and/or host-derived proinflammatory cytokines, E-selectin is transiently expressed on ECs within 2–8 h (Cummings and Smith 1992; Ganesh et al. 2021).

We previously described the role of different classes of carbohydrate ligands on TCs for static binding vs. dynamic adhesion to recombinant murine vs. human E- and P-selectin (Starzonek et al. 2020). We could show that there are indeed species-specific selectin ligands on human cancer cells that are also functional under different binding/ adhesion conditions. Moreover, there seemed to be many more poorly characterized ligands than assumed until now. Therefore, we aimed to comprehensively analyze the different classes of pro-adhesive ligands on TCs in a more physiologic context by using cytokine-stimulated human umbilical vein endothelial cells (HUVECs) instead of recombinant selectins in a laminar flow adhesion assay. Again, we categorized nine human TC lines into three groups according to their canonical E-selectin ligands status, i.e. the presence or absence of sLeA and/or sLeX (Starzonek et al. 2020). The functional relevance of cell surface glycostructures like terminal sialic acids, cell surface glycoproteins, and glycoprotein-bound *N*- vs. *O*-glycans for endothelial adhesion was analyzed by different TC treatments prior to the laminar flow adhesion assay. Adhesive events were distinguished into firm adhesion, rolling, and tethering according to the binding strength.

## Results

### Expression of endothelial selectins and CAMs on HUVECs under basal and stimulated conditions

**Fig. 1:**
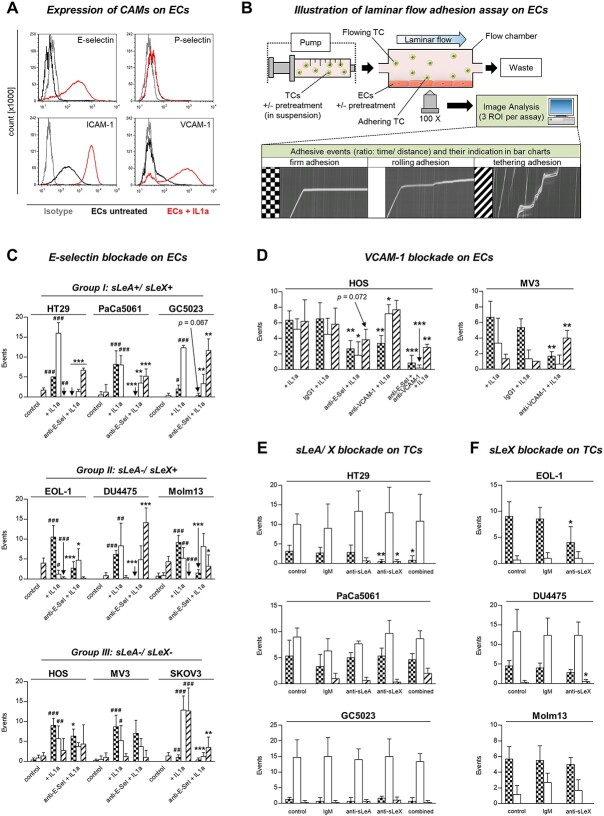
Cell surface expression of CAMs on HUVECs, experimental setup of flow adhesion assay, and effects of antibody blockade on dynamic tumor-endothelium interaction. (A) Flow cytometric determination of CAM expression levels on HUVECs under untreated (black line) and IL1a-stimulated (red line) conditions. Grey lines indicate isotype control measurements. (B) The dynamic adhesion of human TCs to human ECs was analyzed in a laminar flow adhesion assay (shear stress = 0.25 dyn/cm^2^). Adhesive events were captured by video microscopy and distinguished into firm, rolling and tethering adhesions during analysis. These different adhesive interactions were indicated by different bar patterns in all figures as illustrated. EC = endothelial cell, TC = tumor cell, ROI = region of interest. (C) Number of adhesive events/ min of TCs grouped according to their sLeA/X status (group I–III) on ECs. ECs were left untreated or treated with IL1a with or without E-selectin blockade. ^#^*P* < 0.05, ^##^*P* < 0.01, ^###^*P* < 0.001 (IL1a vs. control); ^*^P < 0.05, ^**^P < 0.01, ^***^  *P* < 0.001 (anti-E-Sel + IL1a vs. IL1a). (D) Number of adhesive events/min of TCs on ECs treated with cytokine, E-selectin-, and/or VCAM-1-blocking antibody or isotype control, as indicated. ^*^P < 0.05, ^**^P < 0.01, ^***^  *P* < 0.001 (anti-E-selectin/anti-VCAM-1 + IL1a vs. IgG1 + IL1a). (E + F) number of adhesive events/min of TCs from group I and II on ECs after antibody blockade of sLeA and/or sLeX on TCs. ^*^P < 0.05, ^**^P < 0.01 (anti-sLeA/X-treated vs. IgM). TCs without addition of antibody or IgM served as control. Bars in (C–F) represent mean ± SD of triplicate recordings each from two independent experiments (*n* = 6).

Flow cytometric analyses showed no E-selectin and only low P-selectin expression on ECs under basal conditions, whereas ICAM-1 and, less notably, VCAM-1 were already evidently expressed. Cytokine stimulation of ECs using rhIL1a for 4 h highly upregulated E-selectin, ICAM-1 and VCAM-1, but not P-selectin cell surface expression (Fig. 1A).

### Dynamic adhesion of human TCs with different sLeA/X status on ECs

Nine human malignant TC lines were grouped depending on the TC’s surface selectin ligand status as in our previous study (Starzonek et al. 2020) and were compared for their dynamic adhesion behavior to HUVECs under laminar flow conditions as illustrated in Fig. 1(B): the TC lines HT29, PaCa5061, and GC5023 expressed both canonical E-/P-selectin ligands (group I: sLeA/X-positive). EOL-1, DU4475, and Molm13 cells expressed sLeX only (group II: sLeX-positive). The cell lines HOS, MV3, and SKOV3 lacked both sialyl-Lewis antigens (group III: sLeA/X-negative).

All tested TC lines showed only minor rolling and tethering events on ECs under basal control conditions (Fig. 1C). In contrast, after pretreating ECs with IL1a, all cancer cells could form dynamic adhesions with mostly firm adhesions and rolling events irrespective of the sLeA/X status. GC5023 and SKOV3 were the only cell lines that formed just a few firm adhesions (≤ 3 per minute) and instead more rolling (GC5023) or rolling and tethering (SKOV3) events.

Subsequent E-selectin blockade on ECs (with a function-blocking antibody) drastically affected the adhesion capacity of the cancer cells (Fig. 1C): firm adhesions of all cell lines (and the predominant rolling adhesions in case of GC5023 and SKOV3 cells) were strikingly decreased or completely abolished with an exception for HOS (modest, but significant effect) and MV3 (no effect). These two cell lines additionally (HOS) or primarily (MV3) adhered via VCAM-1 as shown by pre-incubation of ECs with a VCAM-1 blocking antibody (Fig. 1D). The ICAM-1 block had no effect on the adhesion of these two tested cell lines (not shown). The increase in rolling events of HOS cells after VCAM-1 blockade could be abolished by combined antibody blockade of E-selectin and VCAM-1 (Fig. 1D). All sLeA/X-positive cells (group I) additionally showed a decrease in rolling events and an increase in tethering interactions after E-selectin blockade. In the sLeX-positive group (group II), we observed an increase in looser interactions (rolling or tethering) upon E-selectin blockade.

### sLeA and/or sLeX blockade experiments with sLeA/X- and sLeX-positive TCs

Endothelial adhesion of the sLeA/X-positive TCs (group I) was largely unaffected by sLeA and/or sLeX blockade using specific antibodies (Fig. 1E). Only HT29 cells showed a significant reduction in firm adhesions after sLeX and combined sLeA/X blockade, but not after sLeA blockade alone. In case of the sLeX-positive group (group II), sLeX blockade reduced firm adhesions of EOL-1 by ~50 percent (Fig. 1F). The other two cell lines showed almost no change in their adhesive behavior upon sLeX blockade.

### Dynamic endothelial adhesion after neuraminidase treatment of the TCs

**Fig. 2:**
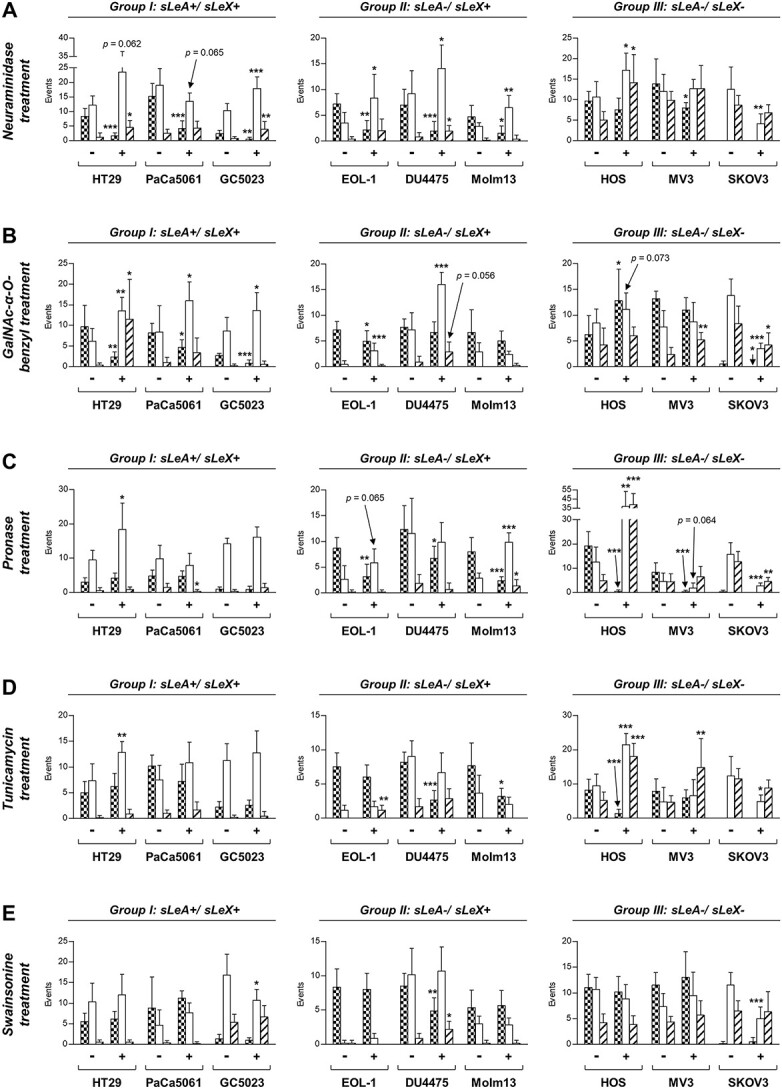
Dynamic endothelial adhesion of TCs after various pre-treatments. Number of adhesive events/min of TCs from groups I to III after TC pre-treatments (+) compared with control treatments (−) as follows: (A) enzymatic cleavage of neuraminic acid residues with *V. cholerae* neuraminidase; (B) inhibition of *O*-GalNAc-glycosylation with GalNAc-α-*O*-benzyl; (C) enzymatic cleavage of cell surface glycoproteins using *S. griseus* pronase; (D) inhibition of *N*-glycosylation in the endoplasmic reticulum (ER) using tunicamycin; (E) inhibition of *N*-glycosylation in the Golgi apparatus using swainsonine. For distinction of adhesive events and corresponding bar patterns, see Fig. 1(B). Stated significance levels (^*^-^***^) as in Fig. 1, referring to treated (+) vs. untreated (−) TCs. Bars in (A–E) represent mean ± SD of triplicate recordings each from two independent experiments (*n* = 6).

The used *Vibrio cholerae* neuraminidase treatment cleaved α2,3-sialic acid, but not α2,6-sialic acid as shown by flow cytometric detection of *Maackia amurensis* lectin-II (MAA-II) and *Sambucus nigra* agglutinin-I (SNA-I) binding to the TC surface (Supplementary Fig. S1A). This treatment commonly and drastically affected dynamic endothelial adhesion of all three TC subsets: as shown in Fig. 2(A), endothelial adhesion of the sLeA/X- and sLeX-positive TCs was largely reduced. For all cell lines of these groups, we observed a significant reduction in firm adhesions with also slightly decreased rolling events of PaCa5061 cells (*P* = 0.065) and increased looser adhesive events in case of HT29 (rolling: *P* = 0.062), GC5023, EOL-1, DU4475, and Molm13 cells. Within the sLeA/X-negative group, MV3 and SKOV3 cells also showed a significant decrease in their respective firmer mode of adhesion (firm adhesion and rolling, respectively), whereas HOS cells remained capable of forming firm adhesions despite neuraminidase treatment (although they developed more loose interactions).

### Dynamic endothelial adhesion after GalNAc-α-O-benzyl treatment of the TCs

GalNAc-α-*O*-benzyl treatment (inhibiting *O-*GalNAc-glycosylation) consistently reduced the number of firm adhesions and increased the number of looser adhesions of all cell lines in the sLeA/X-positive group (Fig. 2B). In contrast, endothelial adhesion of the sLeX-positive cell lines was only marginally (but significantly) reduced in case of EOL-1 cells with decreased firm adhesions and an according increase in rolling events. For DU4475 cells, we observed a significant increase in rolling and an almost significant increase in tethering events (*P* = 0.056). Adhesion of Molm13 cells remained unaltered upon GalNAc-α-*O*-benzyl. In the sLeA/X-negative group, all adhesion forms of SKOV3 cells were significantly impaired, whereas MV3 cells increased their tethering interactions and HOS cells showed significantly increased firm adhesions and by trend more rolling interactions (*P* = 0.073). The chosen GalNAc-α-*O*-benzyl treatment protocol reliably increased the cell surface Tn antigen levels as shown by flow cytometric detection of *Vicia Villosa* (VVA) lectin binding to the TC surface after de-sialylation (representative examples for one cell line per group shown in Supplementary Fig. S1B).

### Dynamic endothelial adhesion after pronase treatment of the TCs

*Streptomyces griseus* pronase treatment (non-specifically cleaving glycoproteins from the cell surface) did not impair endothelial adhesion of sLeA/X-positive cell lines (Fig. 2C). HT29 cells even showed significantly more rolling events without concurrently reduced firm adhesion. In contrast, sLeX-positive and sLeA/X-negative cells were strongly affected. Firm adhesions were significantly decreased upon pronase in case of EOL-1, DU4475, and Molm13 cells, respectively, and were even abolished in case of HOS and MV3 cells. Further, more loose adhesions (rolling and tethering) were observed for Molm13 and HOS cells. Binding of SKOV3 cells was also convincingly reduced upon pronase treatment with a significant decrease in rolling and tethering events. The chosen pronase treatment protocol caused a remarkable overall loss of membrane proteins as detected by Coomassie stainings (Supplementary Fig. S1E).

### Dynamic endothelial adhesion after tunicamycin or swainsonine treatment of the TCs

Tunicamycin treatment (inhibiting *N-*glycosylation initiation in the ER) only partially affected endothelial adhesion (Fig. 2D). Among all sLeA/X-double-positive cell lines, effects were mainly visible in case of HT29 cells showing more of the rather loose rolling interactions after tunicamycin treatment. In the sLeX-positive group, two of the tested cell lines (DU4475, Molm13) showed a significant decrease of the firm adhesions. Moreover, firm adhesions were strikingly diminished in case of HOS cells upon tunicamycin causing a switch from firm to loose (rolling and tethering) interactions. Rolling events of SKOV3 cells were also significantly reduced, whereas MV3 cells showed an increase in tethering interactions. The efficacy of the tunicamycin treatment on *N*-glycosylation was validated by quantifying the amount of mannose residues at the TC surface using Concanavalin A (ConA, Supplementary Fig. S1C).

Swainsonine treatment (inhibiting *N*-glycan maturation in the Golgi apparatus) also only partially affected endothelial adhesion (Fig. 2E). Concerning the sLeA/X-double-positive group, GC5023 cells showed significantly less rolling upon swainsonine. Endothelial adhesion of sLeX-positive cells was largely unaltered except for DU4475 cells showing significantly reduced firm adhesions and instead more tethering events. Both myeloid leukemia cell lines (EOL-1, Molm13) remained unaffected by swainsonine treatment. In the sLeA/X-double-negative subset, we observed a significant decrease in rolling events of SKOV3 cells. The swainsonine treatment was validated to reduce *N*-glycan maturation by means of β1,6-*N*-acetylglucosamine branching and poly-*N*-acetyllactosamine elongation as determined by *Phaseolus vulgaris* leucoagglutinin (PHA-L) and *Datura stramonium* lectin (DSL) binding to the TC surface, respectively (Supplementary Fig. S1D).

Table 1 summarizes the effects of the TC treatments on sLeA/X expression (as published in (Starzonek et al. 2020) and endothelial adhesion (% of control) to visualize the putative correlation between changes in sLeA/X expression and concurrent changes in adhesion.

**Table 1:** Synopsis of the effects of the TC treatments on sLeA/X expression and endothelial adhesion.

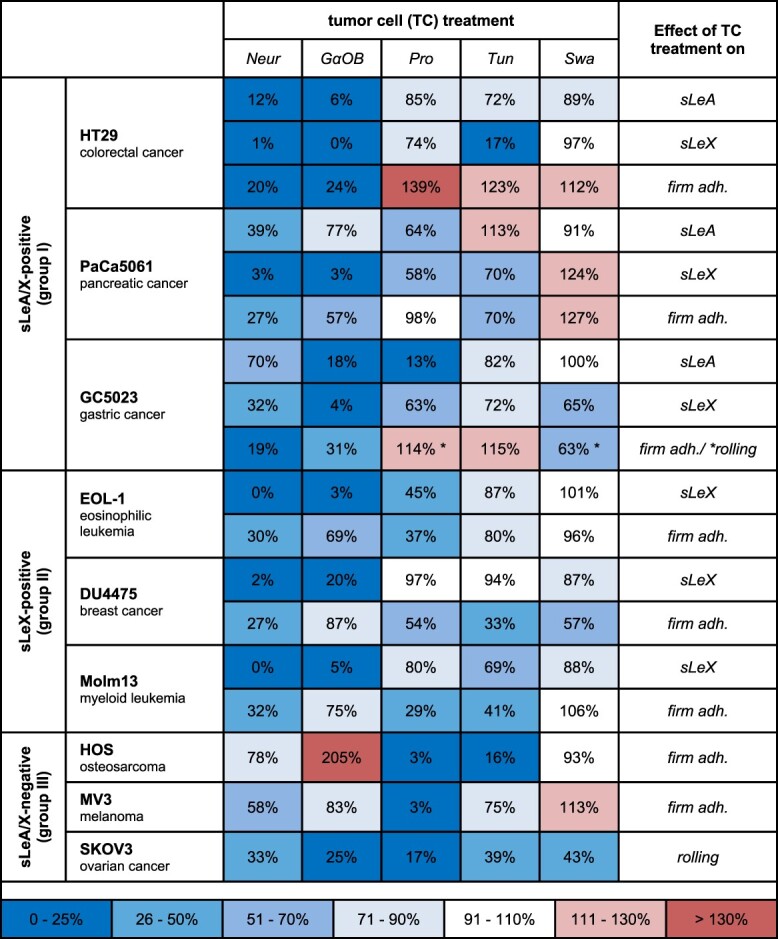

Changes in sLeA and/or sLeX cell surface expression (as published before, Starzonek et al. 2020) and in the number of events/min of the strongest observable adhesion type of each TC line after TC treatment vs. control (100 percent) (see the explanation of colors below). *In the swainsonine and pronase experiments with GC5023 cells, the strongest observable adhesion type was rolling instead of firm adhesion. TC = tumor cell; Neur = neuraminidase; GαOB = GalNAc-α-O-benzyl; Pro = pronase; Tun = Tunicamycin; Swa = Swainsonine; adh. = adhesion.

### Putative pro-adhesive, non-canonical ligands on SKOV3 cells

**Fig. 3:**
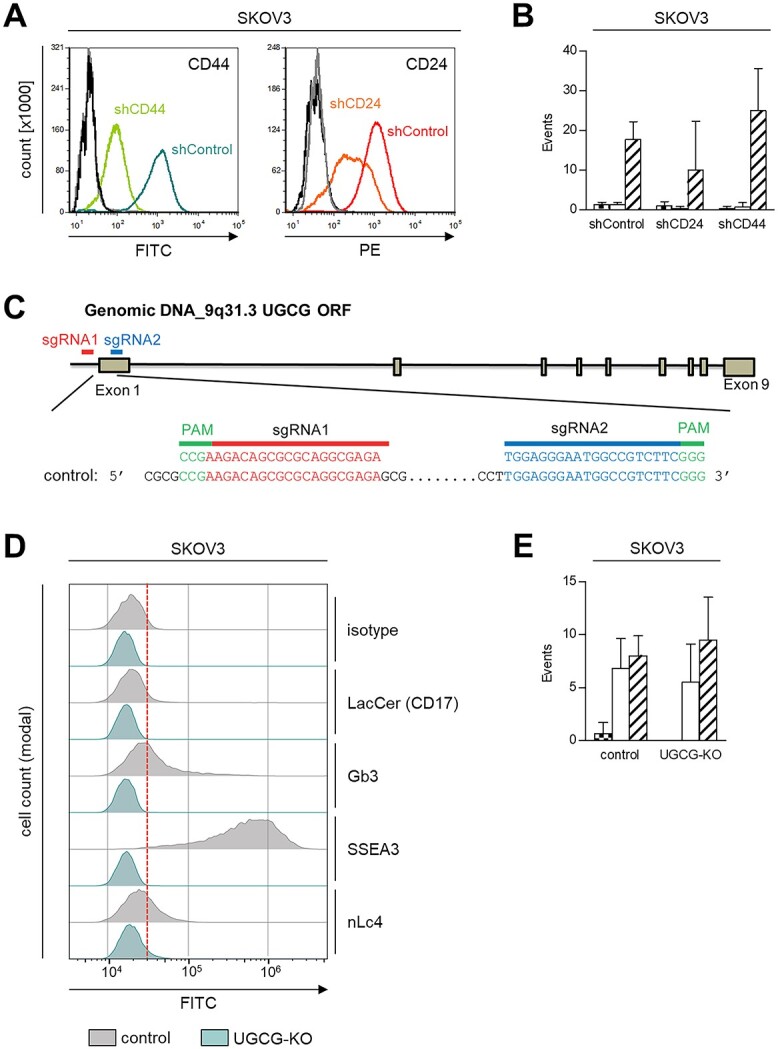
Putative pro-adhesive ligands on SKOV3 cells. (A) CD44 and CD24 knockdown levels on SKOV3 cells determined by flow cytometry. (B) The number of adhesive events/min of SKOV3 cell line derivatives as indicated on IL1a-treated ECs under laminar flow conditions. (C) Strategy to generate a stable UGCG-knockout in SKOV3 using CRISPR-Cas9. (D) Flow cytometric detection of the cell surface glycosphingolipids CD17, Gb3, SSEA3, and nLc4 on control vs. UGCG-knockout SKOV3 cells and (E) corresponding adhesive events/min of these cells on ECs treated with IL1a for 4 h. Bars in (B + E) represent mean ± SD of triplicate recordings.

SKOV3 cells most clearly depended on E-selectin for endothelial adhesion among group III, despite being sLeA/X-negative, and were therefore of particular interest for the identification of novel, non-canonical E-selectin ligands. As the adhesion pattern of SKOV3 cells was sensitive to any of the chosen treatments, it was reasonable to suspect sialylated *N*- or *O*-GalNAc glycans on glycoproteins in the first instance. Thus, we generated SKOV3 cells with stable shRNA-mediated knockdown of CD44 or CD24 (Fig. 3A). However, endothelial adhesion was not altered (Fig. 3B). To exclude glycosphingolipids as potential mediators of adhesion, we additionally knocked out the key enzyme of glycolipid synthesis, i.e. UDP-glucose ceramide glucosyltransferase (UGCG), leading to notable changes in cell surface expression of selected glycosphingolipids (binding sites of sgRNAs illustrated in Fig. 3C). Lactosylceramide (LacCer, CD17), the precursor for most classes of glycosphingolipids, as well as Gb3, SSEA3, and nLc4 were drastically reduced or abolished on UGCG-KO cells compared with control cells (Fig. 3D). Nevertheless, dynamic adhesion on ECs was again not impaired (Fig. 3E).

### Transcriptomic comparison of the tested cell lines focusing on glycosyltransferases involved in sLeA/X synthesis

**Fig. 4:**
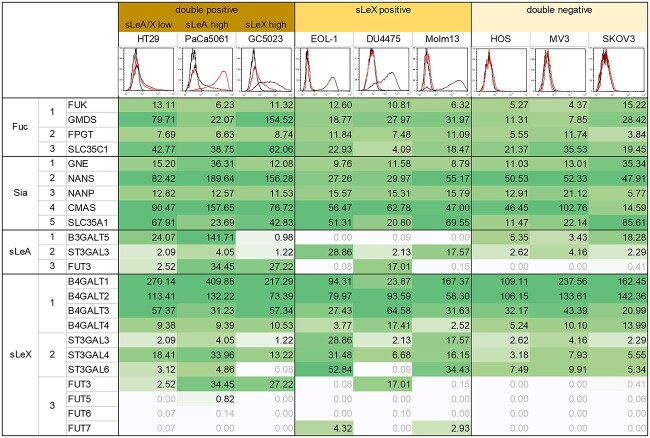
Transcriptomic profiling of glycosyltransferases involved in sLeA/X synthesis. Embedded histograms represent the sLeA/X status of the tested TCs from group I to III. Red and brown lines in histograms indicate cell surface expression levels of sLeA and sLeX, respectively (data from Starzonek et al. 2020 collected again for clarity). Values represent gene expression levels (RNA seq) of glycosyltransferases mediating steps 1–3 of activated fucose assembly (Fuc), steps 1–5 of activated sialic acid assembly (Sia), and steps 1–3 of sLeA or sLeX synthesis. Grey values are below background threshold (cut-off: 0.5). High, low and absent expression levels of glycosyltransferases of the single steps are further illustrated in Supplementary Fig. S1.

In the next step, we investigated whether the chosen grouping of the tested TC lines according to their sLeA/X status (see histograms in Fig. 4) might be helpful to identify key enzymes of sLeA/X synthesis based on glycosyltransferase expression levels. RNA sequencing of TCs from conventional cell culture revealed that all nine cell lines showed notable expression of genes relevant for the stepwise assembly of activated monosaccharides fucose and sialic acid [rows “Fuc” (steps 1–3) and “Sia” (steps 1–5) in Fig. 4] (Duarte et al. 2017). Interestingly, however, although all sLeA/X-double positive cell lines showed detectable expression of key enzymes for both type 1 (Galβ1,3GlcNAc) and type 2 (Galβ1,4GlcNAc) chains (B3GALT5 and B4GALT1-4, respectively), all sLeX-single-positive cell lines lacked B3GALT5 expression (step 1 in type 1, i.e. sLeA synthesis, Fig. 4). All double-negative cell lines (group III) expressed detectable levels of B3GALT5 and B4GALT1-4 but lacked FUT3 and FUT7 expression (step 3 in sLeA and sLeX synthesis, Fig. 4).

In more detail, HT29 cells (showing the weakest sLeA and sLeX expression within the sLeA/X double positive subset) were found to express relatively low levels of FUT3 (Fig. 4). PaCa5061 cells (with the strongest sLeA expression within the subset) were characterized by particularly high B3GALT5 and FUT3 levels (Fig. 4). GC5023 cells (with particularly high sLeX expression within the subset) were found to express comparably low B3GALT5, but high B4GALT1-4 and FUT3 levels. The two leukemia cell lines from the sLeX-positive subset where the only ones with convincing FUT7 expression but lacked FUT3.

Supplementary Fig. S1 further graphically summarizes the overall expression levels of glycosyltransferases relevant for the single steps of sLeA/X synthesis.

## Discussion

Understanding the mechanisms of the physical translocation of TCs from the PT to distant sites is required for the future development of anti-metastatic therapies. In particular, extravasation of TCs from the circulation and the role of E-selectin therein are of crucial importance in this context as they represent key steps in the metastatic cascade (Reymond et al. 2013; Dasgupta et al. 2017). Extending our previous study on the interaction of human TCs with recombinant human vs. murine E- and P-selectin under static vs. dynamic conditions (Starzonek et al. 2020), we herein analyzed the dynamic TC-EC interaction, again considering sLeA/X-positive, sLeX-positive, and sLeA/X-negative TCs (three cell lines each).

Interestingly, all tested TC lines could form various types of dynamic adhesions on stimulated ECs irrespective of their sLeA/X status. The firm adhesions of all TCs from group I and group II on ECs were also observed on recombinant human E-selectin (rhESel) (Starzonek et al. 2020). The only exception were the GC5023 cells, which were partly rolling and partly firmly adhered on ECs but showed firm adhesions on rhESel. This analogy between both studies already suggests a strong relevance of E-selectin for endothelial adhesion of all sLeA/X- and sLeX-positive TC lines, which was additionally confirmed by E-selectin blockade in the present study. The anti-adhesive effect of the used antibody was most likely not due to sterical blockade of other relevant adhesion molecules since shRNA-mediated depletion of E-selectin in HUVECs also drastically reduced endothelial adhesion of the same TCs in a previous study (Lange et al. 2022).

SKOV3 cells were not able to form proper firm (but rolling) adhesions on ECs (reflecting their behavior on rhESel) and clearly showed E-selectin-dependent adhesion. This finding again confirms that E-selectin ligands are not restricted to sLeA and sLeX as widely assumed and that other ligands lacking these glycan epitopes (“non-canonical ligands”) must exist on human TCs (St Hill 2011; Reymond et al. 2013; Starzonek et al. 2020). The other two sLeA/X-negative TC lines from this subset (HOS, MV3) were even able to form firm adhesions on ECs, despite lacking sLeA and sLeX. However, only rolling and tethering events had been observed with these cell lines on rhE-Sel (Starzonek et al. 2020). We therefore analyzed whether other CAMs are involved in endothelial adhesion of these two sLeA/X-negative cell lines which could be confirmed by blocking VCAM-1 on the ECs. This additionally or primarily VCAM-1-dependent adhesion of HOS and MV3 cells, respectively, might contribute to the enhanced adhesion strength of HOS and MV3 cells (firmly adhering on ECs) as compared with SKOV3 cells (rolling on ECs). Thus, (i) non-canonical ligands other than sLeA and sLeX and (ii) co-operation of E-selectin with other CAMs such as VCAM-1 should be considered in the development of therapeutics targeting TC-EC interaction such as glycomimetics (Esposito et al. 2019; Devata et al. 2020). One limitation of the present study is that the VCAM-1 ligands expressed on HOS and MV3 cells were not analyzed (we assume the presence of integrin subunit α4 on these TCs (Dean et al. 1993)). Additionally, the E-selectin blockade experiment was not sufficient to distinguish whether E-selectin mediates firm adhesion directly or whether the blockade rather prevented E-selectin-mediated rolling as an initial event of a cascade, through which firm adhesion is subsequently mediated via VCAM-1/ICAM-1–ligand interactions (Reymond et al. 2013; Sökeland and Schumacher 2019). The experiment with HOS cells and the combined VCAM-1 and E-selectin blockade provides strong evidence that dynamic endothelial adhesion of human TCs can be mediated by such cascade. Moreover, our experimental setup was not suitable to induce cell surface P-selectin expression on ECs so that its potential role was neglected here. Furthermore, HUVECs do not represent an organ-specific microvascular endothelium and might therefore have limited relevance in metastasis research. However, targeting approaches leading to impaired adhesion on HUVEC were commonly closely related to impaired metastasis formation in vivo in a previous study (Lange et al. 2022).

Although adhesion of most of the tested TCs was largely determined by E-selectin, many of them still adhered after sLeA and/or sLeX blockade. Hence, also from these experiments one could conclude the presence of functional, non-canonical E-selectin ligands, even on TCs that concurrently express sLeA and/or sLeX. However, in the absence of such an expected effect, it must be questioned whether the used antibody blocks the functional binding site. As sLeA/X represent relatively small glyco-epitopes in comparison to the antibody, this appears likely. Accordingly, the use of the antibodies did lead to the expected effect in a few of the tested cell lines. For instance, HT29 cells most likely adhere via both sLeA and sLeX. Blocking experiments with combined anti-sLeA and anti-sLeX reduced firm adhesions of HT29; moreover, the striking effects of neuraminidase and GalNAc-α-*O*-benzyl treatments on the sLeA/X levels on HT29 cells were reliably reflected by reduced adhesion. For PaCa5061, the effects of neuraminidase and GalNAc-α-*O*-benzyl indicated a relevance of sLeX for endothelial adhesion or again a combination of sLeA and sLeX. However, anti-sLeA and/or anti-sLeX had no effect on dynamic adhesion in this case. Antibody blockade also did not affect adhesion of GC5023 cells, supporting the observation that treatment effects on sLeA/X expression on these cells mostly did not alter adhesion. In group II, at least the E-selectin-dependent adhesion of EOL-1 cells seemed to occur via sLeX as both antibody blockade of sLeX on the TCs and reduction of sLeX through TC treatment reduced firm adhesion. These findings support an earlier study, in which E-selectin was observed to react strongly with glycoconjugates containing the sLeX epitope using transfected Chinese hamster ovary (CHO) cells expressing a cloned human α1,3/4-fucosyltransferase. This cell line gained the ability to adhere to E-selectin-expressing ECs after synthesizing the sLeX antigen (Cummings and Smith 1992). In contrast, DU4475 and Molm13 cells seemed to primarily adhere via “non-canonical” ligands as the reduction of sLeX through TC treatments not always correlated with reductions in endothelial adhesion (e.g. after GalNAc-α-*O*-benzyl) and vice versa (e.g. after tunicamycin and swainsonine). Moreover, the sLeX-block on DU4475 and Molm13 cells was ineffective to reduce adhesion, further supporting this conclusion.

Besides these rather cell line-specific observations, neuraminidase treatment revealed that terminal α2,3-sialic acid largely determined endothelial adhesion of human TCs. All TC lines except HOS showed drastically impaired endothelial adhesion upon cleavage of sialic acid residues (α2,3-linked but not α2,6-linked sialic acid as demonstrated by lectin binding studies). Interestingly, the TCs were considerably more sensitive to neuraminidase treatment in their adhesion to ECs than to rhESel (Starzonek et al. 2020), especially group II. All sLeX-positive TCs showed no effect in their dynamic binding to rhESel upon neuraminidase treatment, but on ECs. This finding strongly supports earlier studies showing that functional E-selectin-mediated adhesion on ECs not only depends on the presence of E-selectin, but also on concurrent cytokine-induced changes in avidity and microtopology of the contact surfaces (Levin et al. 2001). These changes are neglected in the flow adhesion assay on rhESel.

The strongest effects after inhibition of *O*-GalNAc-glycosylation could be observed in the sLeA/X-positive subset (also observable on rhESel) and for EOL-1. GalNAc-α-*O*-benzyl, a competitive acceptor for the β1,3-galactosyltransferase responsible for extending *O*-linked oligosaccharides from the core GalNAc-Ser/Thr, strongly reduced sLeX expression in all double-positive TC lines and EOL-1 and diminished their ability to adhere to endothelium indicating a high relevance of *O*-GalNAc-glycosylated glycoproteins. Kojima et al. could show similar results on the expression of sLeX and binding of E-selectin using HL60, Colo205 and U937 cells (Kojima et al. 1992). In case of DU4475 and Molm13 cells, however, we also observed a drastic reduction in sLeX levels upon GalNAc-α-*O*-benzyl treatment in our previous study (Starzonek et al. 2020), but no clear effect on endothelial adhesion was observed in the present study. Again, adhesion appears to be mediated by glycans without the sLeX epitope also in case of these TCs.

The effects of non-specific cleavage of glycoproteins were very consistent within the three TC groups and increased from subset to subset. Although dynamic adhesion to ECs in the double-positive group remained completely unaltered by pronase, the same treatment notably reduced adhesion of all sLeX-positive cells and even abolished firm adhesions of HOS and MV3 in the sLeA/X-negative group (SKOV3 with drastically reduced rolling events). Therefore, we assume the ligands for endothelial adhesion of sLeX-positive and sLeA/X-negative TCs mainly on glycoproteins. Again, the treatment effect was more clearly visible on ECs than on rhESel (Starzonek et al. 2020), presumably due to the reasons mentioned above. At least for HOS cells we could previously demonstrate that stable depletion of the glycoprotein CD44 (by lentiviral shRNA transfer) as well as CD44 antibody blockade abrogate their dynamic adhesion on ECs (Lange et al. 2022). CD44 on HOS cells was shown to be *N*-glycosylated and sialylated, to carry DSL- and PHA-L lectin-binding sites and to be a substrate of MGAT5. We therefore assumed poly-*N*-acetyllactosamine on β-1,6-branched *N*-glycans on CD44 to be one possible non-canonical E-selectin ligand on HOS cells (Lange et al. 2022). The sensitivity of SKOV3 cells toward any of the chosen treatments hence prompted us to investigate whether shRNA-mediated knockdown of CD44 impairs the adhesion of SKOV3 on ECs, but no reduction of adhesion was observed. In addition, CD24 was considered as it has been described as a carrier of E-selectin ligands (Myung et al. 2011) and contains numerous *O*-glycosylation sites (Motari et al. 2009), which might explain why SKOV3 cells strongly responded to GalNAc-α-*O*-benzyl. However, efficient, shRNA-mediated depletion of CD24 again did not reduce adhesion. Interestingly, upon lentiviral transduction to transfer the shRNAs, the SKOV3 cell derivatives did not show rolling adhesions anymore but tethered instead, which might be due to the interim puromycin selection. In addition, we blocked glycolipid synthesis using a CRISPR/Cas9-mediated knockout of UGCG, but again did not observe any reduction in the endothelial adhesiveness of SKOV3 cells. As paucimannosidic glycoepitopes have been demonstrated to notably mediate TC adhesion of human glioblastoma cells (Becker et al. 2019), targeting paucimannosylation in SKOV3 cells followed by dynamic adhesion assays on ECs might be a promising next step. Moreover, MAA-II lectin precipitation followed by glycopeptidomics analyses might be helpful to identify glyco-conjugates on SKOV3 cells relevant for endothelial adhesion.

The TCs of the sLeA/X-positive subset (group I) showed reduced adhesion upon GalNAc-α-*O*-benzyl but not upon pronase treatment. This observation has been made on rhESel in our former (Starzonek et al. 2020) and on ECs in the present study and represents a putative contradiction since *O*-GalNAc- (mucin type) glycosylation takes place on glycoproteins, which should generally be cleaved by pronase. However, mucin type glycans are supposed to be highly resistant to proteases (Daniel et al. 2020). On the other hand, we were able to demonstrate a remarkable loss of a broad range of proteins after pronase treatment of these cells. Therefore, we again suggest a particular role for glycosphingolipids (GSL) as putative ligands on these sLeA/X-positive TCs, also for adhesion on ECs. As shown earlier, treating TCs with GalNAc-α-*O*-benzyl significantly alters GSL synthesis (Starzonek et al. 2020). The exact mechanism behind this observation is yet to be clarified. GSLs in general, sialylated GSLs (gangliosides) in particular, and even glycolipids bearing the sLeA or sLeX epitope have already been described to bind E-selectin (Cummings and Smith 1992; Burdick et al. 2001; Shirure et al. 2011). It remains to be determined whether sLeA/X are reduced after blocking GSL synthesis pathways in TCs from group I.

In our approach to identify key enzymes of sLeA/X synthesis we could detect B3GALT5 and B4GALT1-4 for step 1 in the synthesis of sLeA and sLeX, respectively, that were relevant in the chosen TC lines. FUT3 and FUT7 are important for the last step of synthesis. FUT5 and FUT6 were expressed in none of the cell lines. FUT enzymes, catalyzing the incorporation of fucose, are altered in sera and tumor specimens of cancer patients. Elevated fucosyltransferase activity is associated with formation of various tumor antigens (Sen et al. 1983; Vajaria and Patel 2017). FUT3 to FUT7, all belonging to the group of α1,3/4-fucosyltransferases, have been reported to synthesize sLeA and sLeX among other Lewis antigens (Miyoshi et al. 2016). This is in line with our observation of FUT3 appearing to be the predominant fucosyltransferase that synthesizes sLeA and/or sLeX in multiple TC lines of group I and II. In myeloid cells however, the most important fucosylating enzyme appears to be FUT7. FUT7 is a leukocyte-specific FUT and was accordingly only found in the two leukemia cell lines EOL-1 and Molm13 from the sLeX-positive subset. Interestingly, all TCs from the sLeA/X-negative subset (group III) expressed all glycosyltransferases required for sLeX synthesis but lacked FUT expression. Therefore, it appears that FUT3 (in solid malignancies) and FUT7 (in leukemic cells) represent rate-limiting enzymes of sLeA/X synthesis and could be useful targets to block sLeA/X synthesis in cancer cells.

In summary, this study demonstrates that TCs lacking the canonical E-selectin ligands sLeA and sLeX adhere by firm, rolling and tethering interactions on ECs in a partially E-selectin-dependent manner. Thus, so far unknown E-selectin ligands exist on human TCs. Nevertheless, some of the tested sLeA/X-negative TCs additionally or fully depend on VCAM-1 expression on the ECs for firm adhesion. Various TC treatments known to decrease sLeA/X levels as well as sLeA/X blockade only partially reduce firm TC adhesion on ECs. α2,3-linked (but not α2,6-linked) sialic acid is commonly required for firm adhesion (rolling adhesion in case of SKOV3 cells) as is FUT3 as well as FUT7 expression for sLeA/X synthesis. Pronase-mediated digestions of membrane glycoproteins decreases no type of adhesion among the sLeA/X-positive TCs, suggesting a functional role for GSLs in this subset.

## Materials and methods

### Cell culture

Human colorectal cancer cells HT29 and human breast cancer cells DU4475 were purchased from ECACC (Porton Down, UK). The human eosinophilic leukemia cell line EOL-1 was purchased from DSMZ (Braunschweig, Germany) and the human ovarian cancer cell line SKOV3 was from ATCC (Manassas, USA). HOS osteosarcoma cells, Molm13 acute myeloid leukemia cells and MV3 melanoma cells (all human) were kindly provided by the Depts. of Pediatric Hematology and Oncology, Oncology, and Dermatology, respectively (all University Medical Center Hamburg-Eppendorf, UKE). The human pancreatic cancer cell line PaCa5061 was provided by the Dept. of General, Visceral and Thoracic Surgery at UKE (Kalinina et al. 2010). The human gastric cancer cell line GC5023 was newly established in a previous study and cultured as described before (Starzonek et al. 2020).

SKOV3 cells were grown in McCoy’s 5A medium containing 10 percent fetal calf serum (FCS), 2 mM L-glutamine and 1 percent penicillin (50 U/ml) and streptomycin (50 μg/ml) (all from Thermo Fisher Scientific). All other cell lines were cultured in RPMI-1640 medium with 2-mM L-glutamine, supplemented with 10 percent FCS and 1 percent penicillin/streptomycin (P/S) (all from Thermo Fisher Scientific), at 37 °C with 95 percent H_2_O-saturated atmosphere and 5 percent CO_2_.

HUVECs were purchased from PromoCell (Heidelberg, Germany) and cultured in EC medium supplemented with 5 percent fetal bovine serum, 1 percent endothelial cell growth supplement, and 1 percent penicillin/streptomycin solution (all from ScienCell, San Diego, USA), under standard conditions for not more than eight passages.

### shRNA-mediated depletion of CD44 and CD24 in SKOV3 cells

CD44 and CD24 mRNAs in SKOV3 cells were stably depleted by lentiviral transfer of corresponding shRNAs. SKOV3 cells were transduced with a pLVX-puro vector from Clontech (Saint-Germain-en-Laye, France) containing the respective shRNA sequence and subsequently selected with puromycin (500 ng/ml). Transduction of parental SKOV3 cells with a pLVX-puro vector containing a firefly luciferase control shRNA sequence led to shControl cells. The efficiency of the knockdown was measured by flow cytometric analysis. The nucleotide sequences of the used shRNAs were as follows: shCD44: 5’-GGCGCAGATCGATTTGAAT-3′; shCD24: 5’-AGGCCAAGAAACGTCTTCT-3′; shControl: 5’-GTGCGTTGCTAGTACCAAC-3′.

### CRISPR/Cas9-mediated knockout of UGCG in SKOV3 cells

Single guided RNAs (sgRNA) were designed using the web tool of UCSC Genome browser (https://genome.ucsc.edu/). sgRNA1: 5’-TCTCGCCTGCGCGCTGTCTT-3′ and sgRNA2: 5’-TGGAGGGAATGGCCGTCTTC-3′ were selected for gene editing of the exon 1 of *UGCG* gene containing the translational start site. Oligo pairs encoding 20 nt targeted sequences with overhangs (both 5′ and 3′) from *BbsI* restriction site were annealed and cloned into either pSpCas9(BB)-2A-GFP (addgene, #PX458) or pSpCas9(BB)-2A-puro (addgene, #PX459). Constructs were transformed into DH5alpha *E. coli* strains and sequences were confirmed by Sanger DNA sequencing using U6-F primer.

To generate the UGCG-KO cell line, SKOV3 cells were grown in a six-well plate (5 × 10^5^ cells/well) for 24 h and transiently transfected using ViaFect® transfection reagent (Promega) with 3 μg of pair sgRNAs containing donor plasmids to generate homozygous *UGCG* deletion. Seventy two hours after transfection, single cell sorting was performed on a BD FACS Aria Cell Sorter (BD Bioscience) sorting for single GFP+ cells into 96-well flat-bottom plates with pre-warmed media. Single cell clones were isolated and characterized by genotyping PCR using forward primer: 5’-CCGCCGGTTTTGATTAGTGC-3′, and reverse primer: 5′- AACGTTTCCCATTCTCGCCT-3′. The PCR conditions were as follows: using thermocycler at 94 °C for 5 min, followed by 35 cycles of 95 °C for 20 s, 55 °C for 20 s, 72 °C for 30 s, and the last elongation at 72 °C for 5 min. The amplicon for control allele is a 589-bp product and a 334-bp product for UGCG knockout allele.

### Laminar flow adhesion assay

The dynamic adhesion of human cancer cells to ECs was analyzed under physiological flow conditions in a laminar flow adhesion assay at a shear stress of 0.25 dyn/cm^2^ (flow rate: 8.5 ml/h; TC suspension: 1 × 10^5^ cells/ml) as previously described (Richter et al. 2011). ECs were seeded and grown to confluent monolayers in ibidiTreat μ-slide IV^0.4^ flow chambers (ibidi GmbH, Germany; 4.5 × 10^4^ cells/chamber). Prior to the flow assay, ECs were left untreated (control) or stimulated with 10 ng/ml recombinant human IL1a (PeproTech, Germany) for 4 h. Data were acquired and evaluated with CapImage software (version 8.6, Dr. Heinrich Zeintl, Heidelberg). Three regions of interest in the flow chamber were analyzed per condition in each experiment. The adhesive events per minute were distinguished based on their binding characteristics into firm adhesion, rolling and tethering as described before (Starzonek et al. 2020).

### Flow cytometry analysis

The expression of cell adhesion molecules (E-selectin, P-selectin, ICAM-1 and VCAM-1) on ECs upon cytokine stimulation, the sLeA and sLeX status on TCs and the expression of CD44 or CD24 on SKOV3 knockdown cells were analyzed by flow cytometry using the CyFlow Cube 8 (Sysmex) analyzer. ECs were stained with fluorescent mAbs (all from eBioscience) against human CD62E (E-selectin, #12-0627-41), CD62P (P-selectin, #12-0626-80), CD54 (ICAM-1, #12-0549-42), and CD106 (VCAM-1, #12-1069-41) at a final concentration of 1 μg/ml. Fluorescence-labeled mouse IgG1 served as isotype control (eBioscience, #12-4714-42). Fluorescent mAbs against sLeA (anti-CA19-9) and sLeX (anti-CD15s) were from Novus Biologicals (NBP2-54349AF488) and BD Bioscience (#563528), respectively (final concentration: 1 μg/ml). Fluorescence-labeled mouse IgM served as isotype control (BioLegend, #401617). SKOV3 knockdown cells were stained with fluorescent mAbs against human CD44 (Diaclone, #852.601.010) or human CD24 (eBioscience, #12–0247-42) at a final concentration of 1 μg/ml. Fluorescence-labeled mouse IgG1 (eBioscience, #11-4714-42 and #12-4714-42) served as isotype control. Data were analyzed with FCS Express 4 Flow software (De Novo Software, Los Angeles, USA). Dead cells were excluded from the analysis by marking them with propidium iodide prior to the flow-cytometric measurement.

Flow cytometry for detection of the selected glycosphingolipids CD17, Gb3, SSEA3, and nLc4 on SKOV3 UGCG knockout cells was performed as described previously (Alam et al. 2015; Cumin et al. 2022).

### Cell treatment

TCs and ECs were pretreated with blocking antibodies to analyze which molecules mediate dynamic tumor/endothelium interactions. Non-fluorescent antibodies against sLeA (anti-CA19–9) and sLeX (anti-CD15s) were from abcam (#ab3982, clone 121SLE) and BD Bioscience (#551344, clone CSLEX1), respectively, and blockade on TCs was made at 40 μg/ml for 30 min on ice. Mouse IgM from Dako (#X0942) was used as isotype control. E-selectin, ICAM-1 or VCAM-1 blockade on ECs was achieved by adding 20, 10, or 30 μg/ml of non-fluorescent mAb against human CD62E (BioLegend, #336004), CD54 (R&D Systems, #BBA3) or CD106 (R&D Systems, #BBA5), respectively, during the last 30 min of the 4 h cytokine stimulation at 37 °C. Mouse IgG1 (Invitrogen, #02-6100) was used as isotype control for ICAM-1 and VCAM-1 blockade.

The carbohydrate composition on the TC surface was altered by using enzymatic (neuraminidase, pronase), chemical (GalNAc-α-*O*-benzyl) or pharmacological (tunicamycin, swainsonine) treatments (Starzonek et al. 2020). Terminal sialic acid-containing sugar residues were cleaved by using 10 mU/ml neuraminidase (from *V. cholerae*, Roche, Germany) in serum-free medium for 1 h under standard culture conditions (control: serum-free conditions for 1 h) (Gebauer et al. 2013; Wicklein et al. 2013). Pronase (a broadly active mixture of proteases from *S. griseus*, Roche, Germany) was used at 1 mg/ml for 45 min at 37 °C under serum-free conditions (Kobzdej et al. 2002; Mondal et al. 2016) to non-specifically cleave glycoproteins (control: serum-free conditions for 45 min). The inhibition of *O*-GalNAc-glycosylation was obtained with 0.6 mg/ml (2 mM) GalNAc-α-*O*-benzyl (Sigma-Aldrich), directly dissolved in FCS-containing culture medium, added to the cells for 72 h under standard conditions (Huang et al. 1992; Byrd et al. 1995; Huet et al. 1998; Zhang et al. 2014) (control: conventional cell culture). The *N*-glycosylation of glycoproteins was inhibited with two substances intervening at different glycosylation steps: blocking *N*-glycan synthesis in the ER was achieved by using 3 μg/ml (3.6 μM) tunicamycin (from *Streptomyces* sp., Sigma) for 24 h (Dufour et al. 2017) (solvent control: DMSO). Synthetic swainsonine (Sigma, Germany) was used at 0.36 μg/ml (2 μM) for 72 h (solvent control: methanol) to block *N*-glycan processing in the Golgi apparatus.

### Validation of effective TC treatments

To determine the effects of the different TC treatments on the cell surface glycan composition, the binding behavior of different lectins was analyzed by flow cytometry. To estimate the activity of the applied neuraminidase treatment protocol, TCs were fixed with 2 percent formalin after treatment (or control) and analyzed with MAA-II ( Vector Lab., #B-1265), which binds to α2,3-sialic acid, and SNA-I (Vector Lab., #B-1305) binding to α2,6-sialic acid. To assess the efficacy of the GalNAc-α-*O*-benzyl treatment, vital cells were analyzed with *Vicia villosa* agglutinin (VVA, Vector Lab., #B-1235) detecting Tn antigen. VVA binding was examined after de-sialylating the TCs with the aforementioned neuraminidase treatment protocol immediately before the lectin binding assay (to unmask Tn antigen). To explore the efficacy of the tunicamycin treatment, vital TCs were analyzed with *Concanavalin A* (Con A, Vector Lab., #B-1005), which binds to mannose, and to assess the swainsonine effect, vital TCs were tested with PHA-L (ector Lab., #B-1115), which binds to β1,6-GlcNAc branches, and with DSL (Vector Lab., #B-1185) binding to poly-*N*-LacNAc. Lectins were prepared for flow cytometry by incubating 1 μg biotinylated MAA-II, SNA-I or VVA, 5- μg biotinylated Con A and 2-μg biotinylated PHA-L or DSL, respectively, with 0.2-μg streptavidin-APC (BD Bioscienes, #554067) in a total volume of 100-μl lectin buffer (Tris-buffered saline +Ca^2+^ +Mg^2+^) per sample for 15 min at 4 °C. Afterwards, TCs were incubated with APC-labeled lectins for 15 min at 4 °C and subsequently analyzed in a CyFlow Cube 8 (Sysmex) flow cytometer. Except for fixed samples (MAA-II/SNA-I), dead cells were excluded by propidium iodide staining immediately before flow cytometric analysis. To control for non-specific lectin binding of VVA, Con A, PHA-L, and DSL, additional lectin preparations were inhibited with 100-mM GalNAc (Sigma-Aldrich), 100-mM mannose (Serva), 50-μg bovine thyroglobulin (Sigma-Aldrich), or chitin hydrolysate (Vector Lab.), respectively, for 1 h (room temperature, rolling) prior to incubation with additional TC samples. In case of MAA-II and SNA-I, additional TC samples were fixed with 2 percent formalin and afterwards treated with periodic acid to control for non-specific MAA-II and SNA-I binding.

Moreover, the efficacy of pronase treatment was examined by sodium dodecyl sulfate polyacrylamide gel electrophoresis (SDS-PAGE) of cell membrane proteins of pronase-treated TCs compared with membrane protein extracts of untreated cells. Membrane proteins were extracted in triplicates from each TC line by using the ProteinExtract® Native Membrane Protein Extraction Kit (Calbiochem, #444810). Then, 30 μg of the protein lysates were separated according to their molecular mass in a 4–12 percent SDS-polyacrylamide gel and stained with Coomassie Blue.

### RNA sequencing and transcriptome analysis

Total RNA was extracted in triplicates from approximately 5 × 10^6^ TCs per cell line at about 80 percent confluence in triplets using the RNA extraction kit from Qiagen (RNeasy Mini Kit, #74104). The cDNA was synthesized using 500-ng total RNA with the cDNA synthesis kit from Quantseq (#015, Lexogen, Vienna, AT). RNA sequencing was conducted on Illumina HiSeqV4 instrument using single-end read mode (50-nt read length) on HiSeq5A (Illumina, San Diego, CA). For normalization, counts per million mapped fragments (FPM/CPM) using column sums of the raw counts were applied. Average values of the triplicates were used for analysis.

### Statistics

Data were analyzed using GraphPad Prism software (version 5.03, GraphPad Software, Inc., La Jolla, USA) and are presented as means ± SD of the adhesive events (flow adhesion assay) of triplicate recordings each from two independent experiments (*n* = 6). Comparisons between groups (antibody blockade vs. isotype control or treated vs. untreated TCs) were evaluated by Student’s *t*-test. Significance levels were defined as follows: */# *P* ≤ 0.05, **/## *P* ≤ 0.01, ***/### *P* ≤ 0.001.

## List of abbreviations

CAM, cell adhesion molecule; CTC, circulating tumor cell; EC, endothelial cell; hESel, recombinant human E-selectin; HUVEC, human umbilical vein endothelial cell; LacCer, lactosylceramide; PT, primary tumor; sLeA, sialyl-Lewis A; sLeX, sialyl-Lewis X; TC, tumor cell.

## Authors’ contributions

S.S., H.M., M.T.H., and S.M. collected and analyzed the data. S.S. and T.L. interpreted the data. V.F., D.W., K.R., Y.L.H., and F.J. established and characterized knockdown and knockout cell lines. U.S. and T.L. designed the study. U.S. and T.L. provided resources and supervised the study. S.S. and T.L. were major contributors in writing the manuscript. T.L. and U.S. revised the manuscript. All authors read and approved the final manuscript.

## Supplementary Material

Supplementary_Figure_S1_Glycobiology_final_cwad061

Supplementary_Figure_S2_Glycobiology_final_cwad061

supplementary_data_Glycobiology_final_cwad061

## Data Availability

The datasets used and/or analyzed during the current study are available from the corresponding author on reasonable request.
