# Supplementary figures and images for: Identification of potential classes of glycoligands mediating dynamic endothelial adhesion of human tumor cells
Source: Glycobiology. 2023 Jul 24;33(8):637–50. doi: 10.1093/glycob/cwad061 (PMC10560084; doi:10.1093/glycob/cwad061)

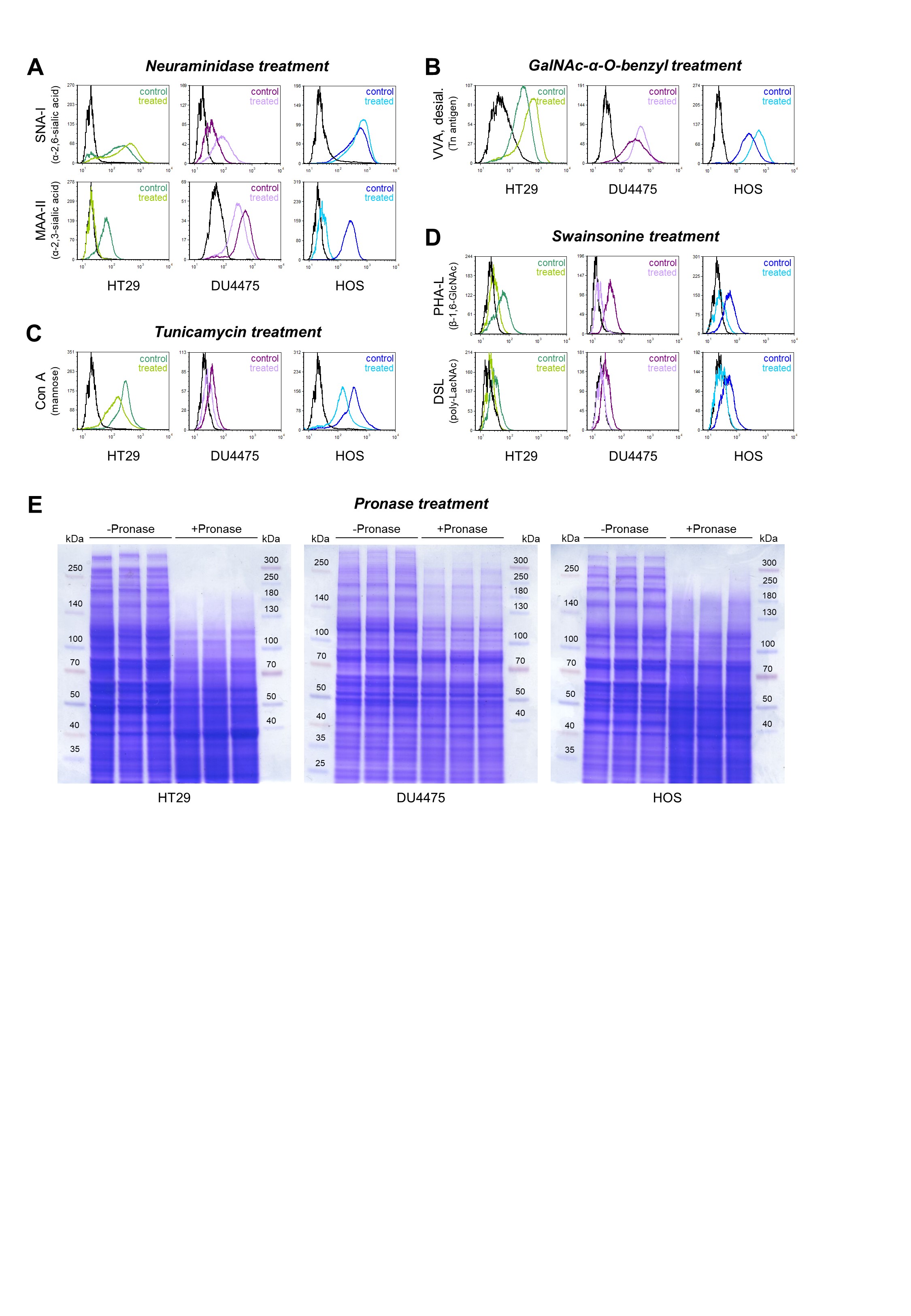

Supplement: Supplementary_Figure_S1_Glycobiology_final_cwad061 [file supplementary_figure_s1_glycobiology_final_cwad061.jpeg]

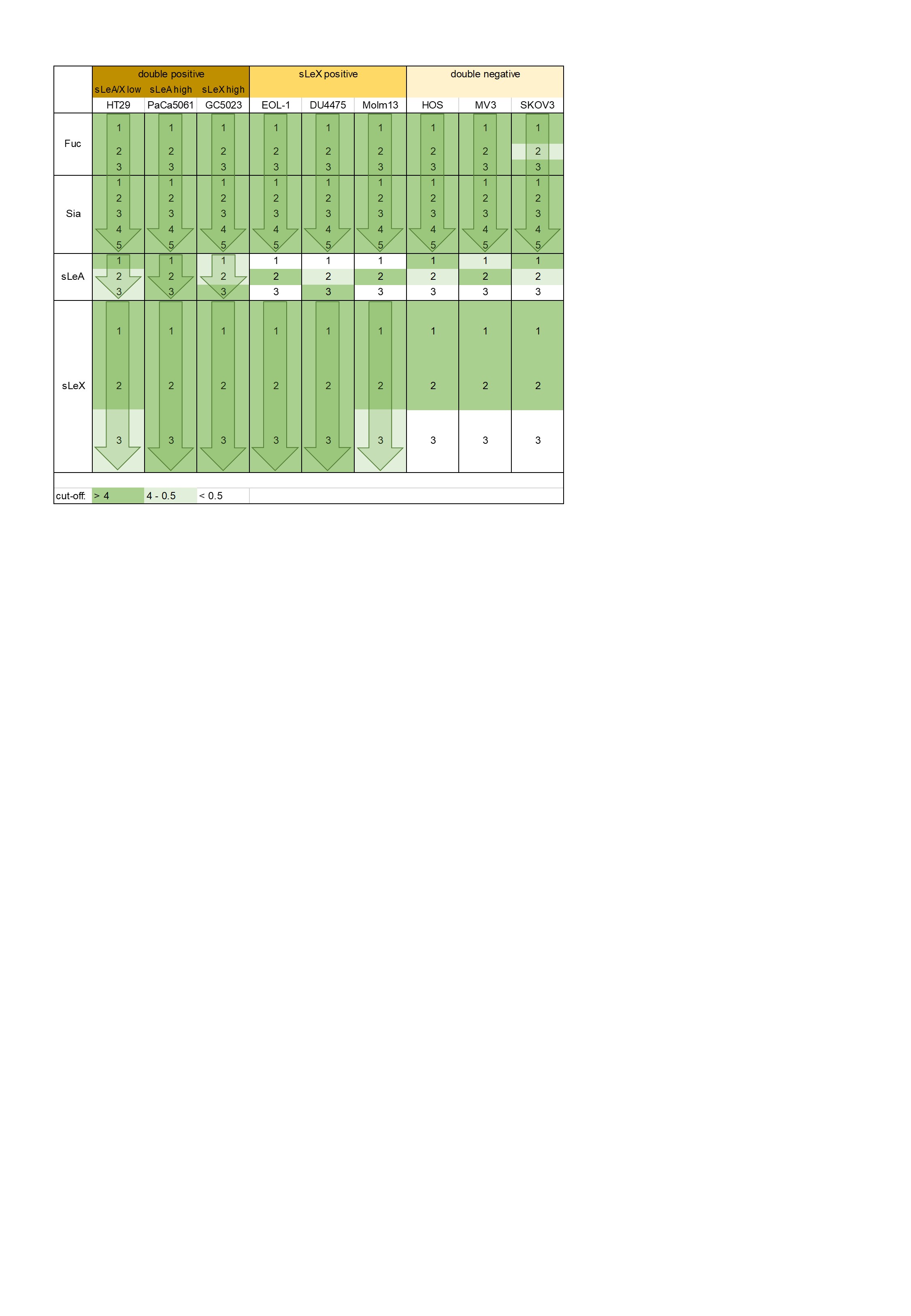

Supplement: Supplementary_Figure_S2_Glycobiology_final_cwad061 [file supplementary_figure_s2_glycobiology_final_cwad061.jpeg]
