# Supplementary material for: Identification of potential classes of glycoligands mediating dynamic endothelial adhesion of human tumor cells
Source: Glycobiology. 2023 Jul 24;33(8):637–50. doi: 10.1093/glycob/cwad061 (PMC10560084; doi:10.1093/glycob/cwad061)
Supplement: supplementary_data_Glycobiology_final_cwad061 [file supplementary_data_glycobiology_final_cwad061.docx]

**Supplementary Figure legends**

**Supplementary Figure S1. Validation of tumor cell treatments.** The efficacy of the applied TC treatment protocols was validated by lectin flow cytometry (**A-D**) and Coomassie Blue-stained SDS-PAGE profiles of membrane proteins (**E**). The cell lines HT29, DU4475 and HOS were considered as representatives of the three TC subsets. The light-colored lines in histograms represent the treated cell sample, the dark-colored lines represent the control sample. **(A)** Enzymatic treatment with *Vibrio cholerae* neuraminidase reduces α2,3-linked sialic acid as indicated by decreased MAA-II binding. **(B)** Inhibition of *O*-GalNAc-glycosylation with GalNAc-α-*O*-benzyl increases Tn antigen (truncated *O*-glycan) levels (VVA binding). **(C)** Inhibition of *N*-glycosylation initiation in the ER by tunicamycin reduces mannose residues at the TC surface (ConA binding). **(D)** Inhibition of *N*-glycan maturation in the Golgi apparatus by swainsonine reduces β1,6-*N*-acetylglucosamine (β1,6-GlcNAc) branching (PHA-L binding site) and poly-*N*-acetyllactosamine (poly-LacNAc) elongation (DSL binding site). **(E)** Enzymatic treatment with *Streptomyces griseus* pronase non-specifically cleaves glycoproteins from the TC surface demonstrated by overall loss of membrane protein bands. Especially large proteins disappeared in favor of blurred bands in the bottom part of the gels.

**Supplementary Figure S2. High, low and absent expression of glycosyltransferases mediating single steps of sLeA/X synthesis.** Graphical summary of the gene expression data from Fig. 4. The glycosyltransferase expression levels of the nine human cancer cell lines are shown in a schematic overview with three colored grades based on cut-offs as indicated. SLeX-positive cells lack the enzyme relevant for step 1 in sLeA synthesis (B3GALT5). SLeA/X-negative cells only lack the enzymes relevant for step 3 in sLeA/X synthesis, i. e. fucosylation.
